# FIV establishes a latent infection in feline peripheral blood CD4+ T lymphocytes *in vivo *during the asymptomatic phase of infection

**DOI:** 10.1186/1742-4690-9-12

**Published:** 2012-02-07

**Authors:** Brian Murphy, Natasha Vapniarsky, Chad Hillman, Diego Castillo, Samantha McDonnel, Peter Moore, Paul A Luciw, Ellen E Sparger

**Affiliations:** 1 Department of Pathology, Microbiology & Immunology, School of Veterinary Medicine, University of California, Davis, 4206 Vet Med 3A, Davis, CA 95616, USA; 2 Department of Pathology and Laboratory Medicine, Center for Comparative Medicine, University of California, Davis, County Road 98 and Hutchison Drive, Davis, CA 95616, USA; 3 Department of Medicine and Epidemiology, School of Veterinary Medicine, University of California, 3115 Tupper Hall, Davis, CA, USA

**Keywords:** Lentivirus, FIV, latency, CD4+CD25+, CD4+CD25-, T cell, monocyte, cat, feline

## Abstract

**Background:**

Feline immunodeficiency virus (FIV) is a lentivirus of cats that establishes a lifelong persistent infection with immunologic impairment.

**Results:**

In an approximately 2 year-long experimental infection study, cats infected with a biological isolate of FIV clade C demonstrated undetectable plasma viral loads from 10 months post-infection onward. Viral DNA was detected in CD4+CD25+ and CD4+CD25- T cells isolated from infected cats whereas viral RNA was not detected at multiple time points during the early chronic phase of infection. Viral transcription could be reactivated in latently infected CD4+ T cells *ex vivo *as demonstrated by detectable FIV *gag *RNA and 2-long terminal repeat (LTR) circle junctions. Viral LTR and *gag *sequences amplified from peripheral blood mononuclear cells during early and chronic stages of infection demonstrated minimal to no viral sequence variation.

**Conclusions:**

Collectively, these findings are consistent with FIV latency in peripheral blood CD4+ T cells isolated from chronically infected cats. The ability to isolate latently FIV-infected CD4+ T lymphocytes from FIV-infected cats provides a platform for the study of *in vivo *mechanisms of lentiviral latency.

## Background

Feline immunodeficiency virus (FIV) infection of cats is an important animal model of human immunodeficiency virus-1 (HIV-1) pathogenesis [1-3]. These two viruses are phylogenetically related [4], and both infect naïve and activated CD4+ T cell subsets as well as monocytes in the susceptible host [2,5,6]. FIV-infected cats develop an acute infection syndrome followed by a prolonged asymptomatic period during which the CD4/CD8 T cell ratio is inverted [5,7]. The asymptomatic phase of infection is generally followed by a terminal immunodeficiency phase of disease termed feline acquired immunodeficiency syndrome (FAIDS), akin to AIDS [7-9].

Latently infected resting CD4+ T cells are the best characterized reservoir for HIV-1 [10]. Such cells are viral DNA-positive and viral RNA-negative and are therefore effectively invisible to pharmacologic therapy and immunological surveillance. The maintenance of latent HIV infection in resting T cells of patients on anti-retroviral therapy (ART) is of serious concern because these cells remain a potential source of virus reactivation [11-13]. Persistence of latently infected memory CD4+ T cells, and potentially other cell types permissive for virus (e.g. macrophages), precludes their elimination by ART or the host immune system for the lifetime of the patient [14] and remains a principal barrier to the long-term pharmacologic and immunologic eradication of lentiviral infections [11,15-18].

For HIV-infected people, there is ample evidence that latently infected reservoirs of CD4+ T cells are established very early on during lentiviral infection, in many cases, prior to the institution of ART [19-26]. Studies have also demonstrated that less than one cell per million resting CD4+T cells from HIV patients on ART harbor latent provirus [10,27]. The study of HIV-1 latency *in vivo *has therefore been hampered by the scarcity of latently-infected cells and restricted access to lymphoid tissues from HIV-infected patients [17,28]. Current models of HIV latency include SIV-infected non-human primates, HIV-infected humanized mice, and a variety of *in vitro *models utilizing either cell lines or primary cells [29]. Although each of these latency models has both advantages and disadvantages, individually each model fails to fully capture the complex properties of HIV latency [29]. Advantages of the primate model include the ability to study multiple different organ systems concurrently and an immune response which resembles a human immune response. Disadvantages of this model include the cost and a "contracted latency phase" relative to HIV-infected humans. Advantages of the mouse model include the ability to model thymic infection and to study infected naïve cells. However, the ability to study specific anatomical sites of infection, such as gut mucosa, is limited; and memory cell infection is more difficult to explore in this model. An additional *in vivo *mammalian model of lentiviral latency would complement latency models currently available. The FIV-infected cat is the only naturally occurring model of lentivirus-induced immunodeficiency. The latency phase of the FIV-infected cat is prolonged and more accurately reflects the time frame of HIV-infected humans prior to the onset of immunodeficiency [30]. The cat's mucosal tissues and immunologic/hematopoietic tissues can be serially and invasively biopsied for evidence of host pathology and virologic changes. Finally, a wide range of immunologic reagents now exists for studying the feline immune system.

This report presents a long-term comprehensive study that defines virologic parameters of FIV infection in isolated peripheral blood leukocytes during the asymptomatic phase and, more broadly, positions the experimentally FIV-infected cat as an animal model of lentiviral cellular latency. Accordingly, viral RNA and DNA was assayed in peripheral blood mononuclear cells (PBMCs), monocytes, CD4+CD25+ (activated/regulatory) and CD4+CD25- (resting) lymphocytes from cats experimentally infected with FIV over an approximately two year observation period. Critical observations revealed persistently detectable viral DNA in all examined cell types whereas viral RNA was not detected in CD4+CD25+ and CD4+CD25- lymphocytes, consistent with the concept of peripheral CD4+ T cell lentiviral latency.

## Results

### FIV infection of cats induced transient lymphadenopathy and alterations in T cell subsets

Four FIV specific pathogen-free (SPF) kittens were inoculated with FIV clade C isolate, FIV-C-Pgmr [31], to assess virus localization and expression over a prolonged time period while two SPF kittens served as mock-infected controls. All inoculated cats seroconverted to FIV by one month post-infection (PI). Mock-infected cats remained seronegative for FIV throughout the study. All FIV-inoculated cats demonstrated bilateral popliteal lymphadenopathy, whereas other peripheral lymph nodes were not affected. Lymphadenopathy manifested as early as three days PI, was generally maintained for approximately 10 months, and was not detected in mock-inoculated control cats. A single cat (165) exhibited periodic, recurrent suppurative paronychia and dorsal cervical/auricular/chin dermatitis. Multiple bacterial and fungal cultures were negative for fungi and intermittently positive for *Streptococcus canis *and *Escherichia coli*. These inflammatory lesions emerged at 5-12 months PI and were managed with appropriate topical and oral antibiotic treatments.

**Figure 1:**
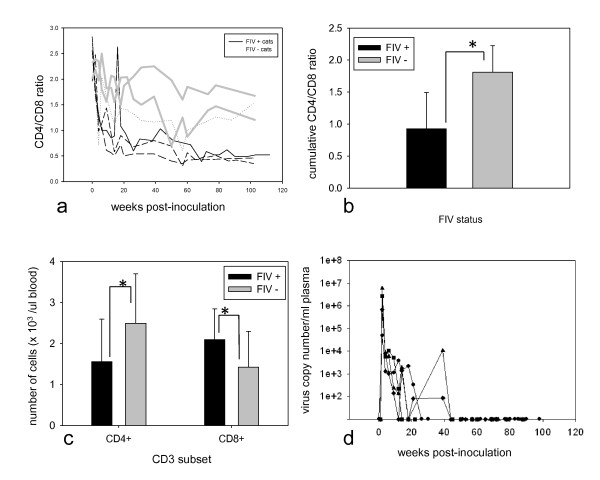
**Persistently inverted CD4/CD8 ratio despite undetectable plasma viral RNA**. The CD4/CD8 T cell ratio for each FIV-infected and uninfected control cat is plotted as a function of time (a). Cumulative data from each of the 2 uninfected control cats are plotted as thick grey lines while data from the 4 individual FIV-infected cats are plotted as solid black lines (165), medium-dashed lines (184), short-dashed lines (186) and dotted lines (187). The cumulative mean CD4/CD8 T-cell ratio for FIV-infected cats (black bar) was derived from all individual CD4/CD8 T-cell ratios obtained from 2 weeks to 112 weeks PI and compared to the ratio for the mock-infected control cats (grey bar). Significance is shown using p value with * p = .0001 (b). Cumulative means for the absolute number of CD4+ and CD8+ T cells for FIV-infected cats were compared to values for uninfected control cats. Statistical significance is marked by an asterisk (*) and for this analysis p = .0017. Error bars denote standard deviation (c). Plasma viral RNA copy numbers based on a FIV *gag*-based real-time PCR assay are plotted for each FIV-infected cat: circle (cat 165), diamond (184), square (187) and triangle (186) (d). Assay detection threshold is approximately 80 copies viral RNA per ml.

The CD4/CD8 T cell ratio for each FIV-infected and uninfected control cat was plotted as a function of time in Figure 1a. Although ratios were variable for both groups of cats, these data suggested that FIV infection resulted in a decrease in the CD4+ T cell subset in blood. Furthermore, the mean value (0.925 +/- 0.567) for cumulative CD4/CD8 T cell ratios from 2 to 112 weeks post infection for FIV-infected cats was significantly less than that for the FIV-uninfected cats (1.8113 +/- 0.412; p < .0001; Figure 1b). Similarly, the cumulative mean value for absolute CD4+ T cell counts in blood (1556/μl blood +/- 1036) for infected cats was lower compared to that measured for uninfected controls (2490/μl +/- 1209, p = .0017; Figure 1c). In contrast, the cumulative mean value (2095/μl +/- 752) for blood CD8+ T cell counts was greater for infected cats compared to uninfected cats (1423/μl +/- 875, p = .0017; Figure 1c).

### FIV infection resulted in a transient plasma viremia

Plasma viral RNA (vRNA) quantification by a real-time reverse transcription (RT)-PCR assay revealed a peak viremia at 2 weeks PI that ranged from 4.9 × 10^4 ^to 5.8 × 10^6 ^copies vRNA/ml of plasma (Figure 1d). Plasma viremia was variably detectable from 2-44 weeks PI. From 44 up to 98 weeks PI, plasma vRNA was not detectable within the limits of the same real-time PCR assay for all FIV-infected cats. The threshold of detection for the vRNA assay was approximately 80 copies vRNA per ml of plasma. Mock-infected control cats were negative for plasma vRNA at all time points tested (data not shown).

### For the examined cell types, viral RNA expression was restricted to monocytes in peripheral blood

To examine the question of persistent latent FIV infection of specific reservoir cell populations in the host, assessment of cell-associated FIV viral DNA (vDNA) and viral RNA (vRNA) for different cellular subsets was critical, particularly in parallel with assay of plasma for detectable vRNA. Therefore, standard methodology involving IgG-coated magnetic beads, a fluorescence-activated cell sorter, and monoclonal antibodies, either specific or cross-reactive for feline cell surface markers, was used to purify CD4+ T cells and monocytes from PBMCs. CD4+ T cells were further purified for subsets that were either CD25+ or CD25- to distinguish activated from resting cells respectively. Mean cell numbers isolated for each subset were 1.8 × 10^7^, 3.6 × 10^5^, 5.5 × 10^4 ^and 1.5 × 10^6^, for PBMCs, monocytes, CD4+CD25+ and CD4+CD25- cells, respectively. Purified populations of CD4+CD25+, CD4+CD25-, monocytes, and PBMCs were next assayed for both viral DNA and RNA loads using real-time PCR assays.

**Figure 2:**
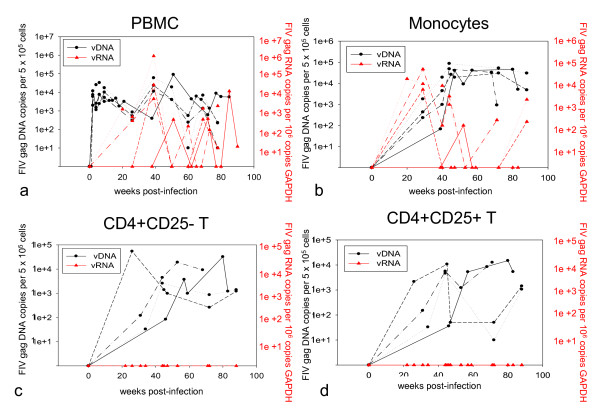
**CD4+ T cells persistently infected in the absence of detectable plasma viral RNA**. PBMCs (a), monocytes (b), CD4+CD25- T cells (c), and CD4+CD25+ T cells (d) isolated from blood from all FIV-infected cats were examined over time PI for viral DNA and vRNA by real-time PCR assays as described in Methods. Data from individual FIV-infected cats are plotted as solid black lines (165), medium-dashed lines (187), short-dashed lines (186) and dotted lines (184).

Viral DNA was detected in PBMCs from all FIV-infected cats at all time points after virus inoculation (Figure 2a). DNA copy number ranged from 10 to 9.0 × 10^4 ^copies/5 × 10^5 ^cells. Viral RNA was intermittently detected in PBMCs of all four FIV-infected cats and ranged from 10 to 9 x10^5 ^copies/10^6 ^copies GAPDH (Figure 2a). The reason for this dramatic shift in vRNA copy number has not been determined but does not seem to be reflected in the clinical health of the cats at that time. In general, *gag *vRNA varies by no more than 2-3 logs from one sample collection time point to the next (generally set 10 weeks apart). Viral DNA (vDNA) was detected in freshly isolated feline monocytes obtained from all FIV-infected cats at all time points PI (Figure 2b) and ranged from 67 to 8.6 × 10^4 ^copies/5 × 10^5 ^cells. Viral RNA was sporadically detected in monocytes derived from all four cats and ranged from 10^2 ^to 4.6 x10^4 ^copies/10^6 ^copies GAPDH. Viral DNA was also detected in both CD4+CD25- and CD4+CD25+ lymphocytes isolated from all FIV-infected cats at all time points after inoculation and ranged from 33 to 5.4 × 10^4 ^copies/5 × 10^5 ^cells or 10 to 1.5 × 10^4^/5 × 10^5 ^cells (Figure 2c and 2d, respectively). Importantly, vRNA was not detected in CD4+CD25- or CD4+CD25+ T cells isolated from FIV-infected cats for any time points tested, although early acute infection time points prior to 20 weeks PI were not tested. These findings revealed a highly restricted pattern of vRNA expression that was suggestive of FIV latency in these vDNA-positive CD4+ T cell subsets during early chronic infection. Viral DNA and vRNA were not detected in PBMCs or any cell preparations isolated from uninfected control cats (data not shown). The threshold of detection was calculated to be approximately 10 copies of FIV gag per 5 × 10^5 ^cells.

### Episomal circular LTR DNA was not detected by standard PCR assays in PBMCs or purified T cell subsets during chronic infection

**Figure 3:**
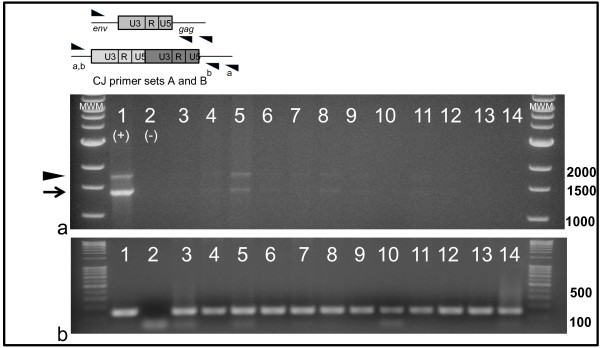
**Analysis of viral circle junctions in cells isolated from chronically infected cats**. Genomic DNA purified from freshly isolated PBMC samples prepared from FIV-infected cats up to 14 weeks PI were tested for viral episomal circular LTR DNA by standard PCR assays (CJ primer set A) designed to detect circular forms of vDNA (a). Circle junction PCR amplicons representative of either single LTR (arrow) or double LTR circles (arrowhead) were generated from *in vitro *FIV-infected feline PBMCs (lane 1; positive control) and were absent in the negative control using a water template (lane 2). Circle junction PCR reactions with expected PCR amplicons of 1423 bp (single LTR, arrow) and faint 1820 bp (double LTR, arrowhead) are shown for PBMCs from cats 184, 186 and 187 sampled at 4 wks PI (lanes 3, 4 and 5); 6 wks PI (lanes 6, 7 and 8); 9 wks PI (lanes 9, 10, 11); and 14 wks PI (lanes 12, 13 and 14), respectively. PCR products from standard PCR reactions for 18s rRNA (b) from samples in the same corresponding lanes in (a), reveal positive, appropriately sized bands (~140 bp) except for lane 2 (water template negative control).

In addition to the linear DNA intermediates formed during the reverse transcription step, two circular forms of viral DNA have been identified in retrovirus-infected cells [32] and are characteristic of a productive viral infection while generally not present in latently infected cells [33]. To further investigate the replication status of viral DNA-positive cells, forward and reverse primers were designed to amplify and detect FIV-C-Pgmr circular episomal DNA products with either a single or double LTR junction (circle junction, CJ). Progressively fainter CJ PCR amplicons (CJ primer set A) were detected in freshly isolated PBMCs from cats 184, 186 and 187 at 4, 6 and 9 weeks PI (Figure 3a). However, by 14 weeks PI, CJ episomal DNA was no longer detected in PMBC. PBMCs isolated from a FIV-infected cat at six weeks PI and cultured *in vitro *for six days served as a positive control revealing both single and double LTR CJ PCR products (Figure 3a). DNAs isolated from monocytes, CD4+CD25+ lymphocytes and CD4+CD25- lymphocytes purified from four different FIV-infected cats from time points extending from 20 to 83 weeks PI were also examined. No CJ PCR products (CJ primer set A) were detected for any sample tested although all samples were positive for 18s rRNA gDNA (data not shown). These results are consistent with a detectable, productive viral infection in PBMCs during acute infection that becomes undetectable during the transition into early chronic infection at 14 weeks PI.

### Ex vivo cultivation of PBMCs harvested from cats during chronic FIV infection results in transcriptional activation and production of infectious virus

**Figure 4:**
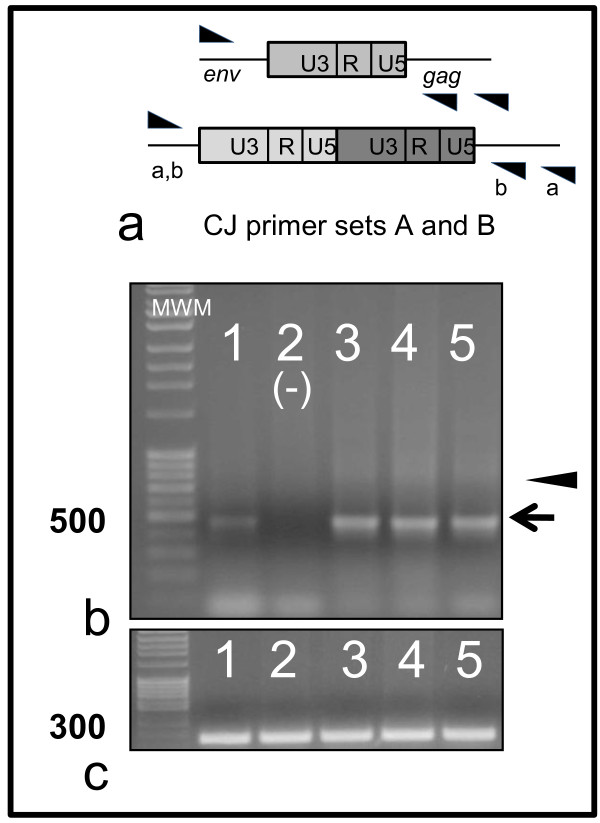
***In vitro *viral rescue assay in PBMC**. Schematic diagrams of circle junction PCR utilizing primers outside the LTR, circle junction primer sets A and B (a) Primers are indicated by triangles. PBMCs were isolated at 50 weeks PI from both FIV-infected and uninfected control cats and cultured *ex vivo *for 10 days followed by PCR amplification for LTR circle junction sequences (CJ primer set B) (b). Expected PCR amplicons of 500 bp (single LTR, arrow) and faint 800 bp (double LTR, arrowhead) are present. Circle junction PCR products generated from cultured PBMCs from cats 184, 185 (FIV negative, negative control), 186, 187 and 165 are represented by lanes 1-5, respectively. PCR products were generated for 18s ribosomal RNA gene (d) from the same samples as in (c). All lanes have positive, appropriately sized amplicons (140 bp) for 18s rRNA.

At 50 and 134 weeks PI, blood was harvested from FIV-C-Pgmr-infected and uninfected cats to determine infectivity of FIV proviruses detected in PBMCs. Genomic DNA preparations from freshly isolated PBMCs were tested for FIV *gag *sequences using real-time PCR. FIV *gag *PCR amplicons were detected in cells isolated from all FIV-infected cats. For the 50 week PI samples, CJ PCR analysis (CJ primer set B) performed on the same DNA samples failed to detect appropriately-sized amplicons by standard PCR assay. Viral gag RNA was not detected by real-time RT-PCR for the same freshly isolated PBMCs harvested from FIV-infected cats (data not shown). To determine if detectable FIV DNA was capable of producing replication competent virus particles, PBMCs isolated from FIV-infected and uninfected cats at 50 weeks PI were also cultured *ex vivo *in PBMC media supplemented with mitogens concanavalin A (Con A) and phorbol myristate acetate (PMA) for 3 days followed by passage in PBMC culture media for 7 additional days as previously described [34]. Genomic DNA samples prepared from *ex vivo*-cultured cells (harvested at 10 days post-cultivation) were subsequently shown to be positive for single (500 bp) and double (faint 800 bp) CJ LTR DNA amplicons (CJ primer set B, Figure 4a) for all four FIV-infected cats while samples from an uninfected control cat were negative (Figure 4b). Although the double CJ LTR amplicon is faint, the lack of a single CJ LTR amplicon in the negative control lane suggests that it lacks a double as well (the single LTR circle amplicon seems to be a favored end-product of the polymerase chain reaction relative to the double LTR as a result of its shorter amplicon size). Furthermore, viral RNA was detected by real-time PCR in supernatants harvested from PBMC cultures, whereas cultures from uninfected control cats were negative for viral RNA (data not shown).

To assess infectivity of *ex vivo *generated virions, clarified PBMC culture supernatants were inoculated onto PBMCs freshly isolated from uninfected SPF cats. At 7 days post-cultivation, whole cell DNA and RNA were isolated from cultured PBMCs, and RNA isolated from culture supernatants were positive for viral *gag *sequences for three of the four FIV-infected cats (184, 165 and 186) when assayed by real-time PCR. Samples derived from an uninfected control cat (185) were negative for viral nucleic acid as expected. These data indicate that *ex vivo *activation of viral RNA-negative PBMCs from FIV-infected cats results in the production of infectious virions.

**Figure 5:**
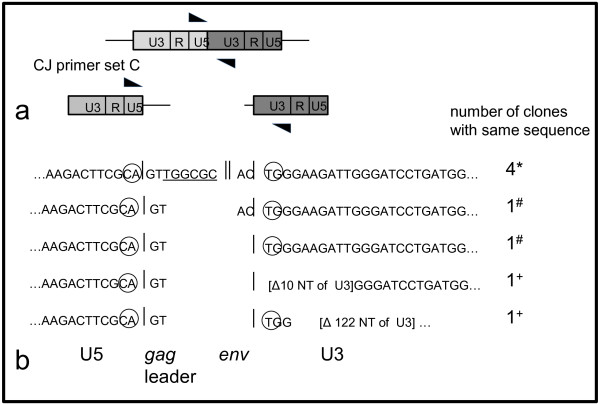
**Cloned viral 2-LTR circle junction sequences**. PBMCs were isolated from FIV-infected cats 6 weeks (184) and 12 weeks (165) PI and cultured *ex vivo*. DNA was isolated from *ex vivo*-cultured cells and 2-LTR CJ PCR was performed (CJ primer set C). 2-LTR CJ PCR products were cloned and sequenced from cat 184 PBMCs incubated *ex vivo *for 3 days (*) and from cat 165 PBMCs incubated 4 (#) and 6 days (+). Circled nucleotides represent the invariant CA and TG dinucleotides within the U5 and U3 regions, respectively, while the underlined nucleotides represent a portion of the primer binding site within the *gag *leader.

Primers designed to amplify LTR CJ located outside of the LTR (i.e. within *gag *and *env*) can utilize linear DNA molecules as substrates, yielding spurious CJ products indistinguishable from genuine CJ amplicons [35]. This process is thought to occur first through linear amplification of the LTR followed by LTR hybridization with subsequent exponential amplification. It is possible, however, to design forward and reverse primers complementary to the U5 and U3 sequences, amplify across the unique LTR-LTR junction and specifically amplify 2-LTR CJ (Figure 4b). Therefore, we designed a real-time PCR assay utilizing 2-LTR CJ primers (FIV_U5 for _and FIV_U3 rev_, CJ primer set C, Table 1). Each real-time PCR assay was run in triplicate followed by a melt curve to assess amplicon validity. Following PCR, agarose gel electrophoresis was performed to assess amplicon size. Two LTR CJ primers (CJ primer set C, Figure 5a) were utilized in a PCR reaction with PBMCs isolated from all four FIV-infected cats and one uninfected cat (185).

**Table 1:** Primers used for PCR amplification analysis

Primer name	Sequence	FIV genomic position *	Assay
feline _GAPDH for_	5' AAA TTC CAC GGC ACA GTC AAG	NA	Feline GAPDH QT PCR

feline _GAPDH rev_	5' TGA TGG GCT TTC CAT TGA TGA	NA	Feline GAPDH QT PCR

18s _rRNA for_	5' GTA ACC CGT TGA ACC CCA TT	NA	Feline 18s rRNA

18s _rRNA rev_	5' CCA TCC AAT CGG TAG TAG CG	NA	Feline 18s rRNA

FIV _QT gag for_	5' TAG CCC TTG ACC CAA AAA TG	1067	FIVgag QT PCR

FIV _QT gag rev_	5' ATT GGC CGA AAA AGC TGT AA	1167	FIVgag QT PCR

FIV _Gag leader for_	5' GTT GGC GCCCGA ACA GGG	354	FIV *gag *sequencing

Gag _circle rev_	5' GTA GAT GGT CTG GTG TCT AAT CCC A	989	FIV *gag *sequencing

FIV _U5 rev_	5' TGC GAA GTC TTC GGC CCG GAC TCC G	338	FIV LTR sequencing

FIV _env for_	5' TGG GAG TCC TCT GAC CGA GA	9043	FIV LTR sequencing + CJ A and B

FIV _gag circle rev3_	5' GGT TTC ACT TTC CTA GCC TGC TC	403	CJ B

FIV _gag rev_	5' TGA GTC AGC CCT ATC CCC ATT A	1345	CJ A

FIV _u5 for_	5' CCT GTC GTG TAT CTG TGT AAT CTT TTC TAA C	290	CJ C

FIV _u3 rev_	5' TGG AAC AAA ATC TAC GTC ATC GG	155	CJ C

QT PCR = real time PCR; CJ = circle junctions PCR; NA = not applicable

* 5' position of FIV primer

DNA samples derived from PBMCs harvested at 9, 28, 40 weeks PI (184), 78 weeks PI (184, 186, 187), 90 and 102 weeks PI (165) were all positive for real-time 2-LTR CJ amplicons. Samples derived from PBMCs harvested at 122 weeks PI (186) and 134 weeks PI (165) were negative for real-time 2-LTR CJ amplicons. In order to assess the validity of the 2-LTR CJ PCR amplicons, samples were cloned from *ex vivo*-cultured PBMCs harvested from two FIV-infected cats. PBMCs were isolated from FIV- infected cats at 6 weeks (184) and 12 weeks (165) PI and cultured *ex vivo *for 3 to 6 days. DNA was isolated from *ex vivo*-cultured cells, 2-LTR CJ PCR was performed (CJ primer set C), and PCR amplicons were cloned and sequenced (Figure 5b). All of the sequenced samples demonstrated intact U5 regions with variable numbers of nucleotides from the *gag *leader (2-8 nucleotides) and *env *open reading frames (0-2 nucleotides) at the unique LTR-LTR circle junction. Six of the clones contained intact U3 regions, while two demonstrated variable blocks of deleted nucleotides from U3.

### Ex vivo cultivation of CD4+ T cells harvested from cats during chronic FIV infection results in transcriptional activation and the production of infectious virus

**Figure 6:**
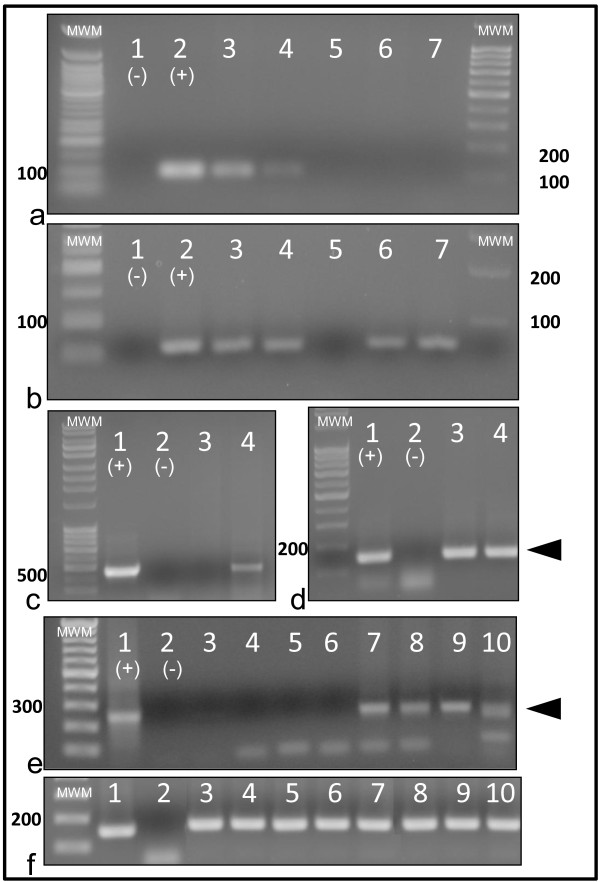
***In vitro *viral rescue assay in CD4+ T cells isolated by magnetic column at 126 weeks PI**. FIV gag PCR products were present in samples from FIV-infected cat (165) vDNA (lane 3) and vRNA (lane 4) (a). Amplicons were not identified in RT- samples (lane 5, cat 165 cDNA), or in samples from the uninfected control cat (cat 183, DNA and cDNA, lanes 6 and 7 respectively). Positive [pDNA (plasmid), lane 2] and negative controls (water template, lane 1) were appropriate. Appropriate-sized amplicons were generated with the feline GAPDH primer set (b) from the same samples as in (a). CD4+ T cells derived from cat 165 were cultured *ex vivo *for 14 days and subsequently co-cultured for 6 days with SPF CD4+ T cells (lane 3, negative) or PBMCs (lane 4, positive) and evaluated for FIV circle junctions (primer set B) (c). Positive (pDNA, lane 1) and negative controls (water template, lane 2) were appropriate. Appropriate-sized amplicons (arrowhead) were generated for 18s rRNA gene (d) from the same samples as in (c). Co-culture of latently infected CD4+ T cells with uninfected feline PBMCs results in detectable 2 LTR circle junction amplicons (e). Freshly isolated CD4+ T cells derived from FIV-infected cats 165, 184, 187 and 186 (day 0, lanes 3-6, respectively) were cultured *ex vivo *for 14 days (165) or 3 days (184, 187 and 186) and subsequently co-cultured for 6 (165), 5 (187 and 184) or 10 (186) additional days with FIV-negative PBMCs (lanes 7-10, respectively). Samples derived from cat 186 PBMCs cultured *ex vivo *for 13 days served as the positive control (lane 1) while water template served as the negative control (lane 2). Expected PCR amplicons of 250 bp were generated with 2 LTR circle junction primer set C in lanes 1 (positive control) and 7-10 (arrowhead). Appropriate-sized amplicons (arrowhead) were generated for 18s rRNA gene (f) from the same samples as in (e).

To determine if viral transcription in latently infected CD4+ T cells isolated from chronically infected cats could be reactivated, we performed *ex vivo *culture experiments with magnetic column-isolated CD4+ T cells. At 124 to 126 weeks PI, blood was harvested from the 4 FIV-infected cats (165, 184, 186 and 187) and an uninfected control cat (183). Genomic DNA preparations from freshly isolated CD4+ T cells were tested for FIV *gag *sequences using real-time PCR. FIV *gag *PCR amplicons were detected in cells freshly isolated from the FIV-infected cats (data not shown) although 2-LTR CJ real-time PCR analysis performed on the same DNA samples failed to detect amplicons (Figure 6e). Primer concatemers are evident in lanes 4-8 and 10 at approximately 100-150 nucleotides, primer concatemers tend to form stochastically in the absence of target template or when the template is limiting. Viral gag RNA was not detected by real-time RT-PCR using the same freshly isolated CD4+ T cells from any cat (data not shown). Although these findings are consistent with viral latency in peripheral CD4+ T cells, due to the inherent limitations in the sensitivity of any test, we cannot absolutely rule out the possibility that low level viral transcription is ongoing in these cells.

CD4+ T cells from FIV-infected and uninfected cats were cultured with ConA and PMA mitogens for 3 (184, 187, 186) or 14 days (165, 183) as described above. Both viral gag DNA and RNA were detected in samples obtained from FIV-infected cat 165 by real-time PCR and appropriately sized amplicons were evident on agarose gel electrophoresis (Figure 6a). Circle junction LTR DNA was not detected at this time point (data not shown). The cultured CD4+ T cells were subsequently co-cultured with an equal number of uninfected feline CD4+ T cells or PBMCs for 5 (187, 184), 6 (165) or 10 (186) additional days. Genomic DNA samples prepared from cat 165 *ex vivo*-cultured CD4+ T cells co-cultured with uninfected PBMCs were subsequently shown to be positive for CJ DNA (CJ primer set B) while cat 165 CD4+ T cells co-cultured with uninfected CD4+ T cells were negative for CJ DNA (Figure 6c). 2-LTR CJ PCR assays (CJ primer set C) performed with DNA isolated from CD4+ T cells from FIV-infected cats co-cultured with uninfected feline PBMCs demonstrated appropriately sized amplicons (Figure 6e) consistent with a productive viral infection (mean real-time PCR C_T _values for lanes 1, 7-10, respectively: 29.9, 36.9, 37.1, 37.2 and 33.9). In addition, magnetic column freshly isolated CD4+ T cells (126 weeks PI) from FIV-infected cat 187 were sorted by flow cytometry in the presence of the CD4 antigen and absence of CD14 antigen. Sorted CD4+ T cells, determined to be 99.6% pure, were cultured *ex vivo *for 2 days followed by co-culture with SPF feline PBMCs for 14 days. Consistent with a productive infection, DNA isolated from the *ex vivo*-cultured cells were positive for 2 LTR circle junctions by real-time PCR (data not shown).

### FIV LTR and gag sequences are relatively stable during chronic infection

**Figure 7:**
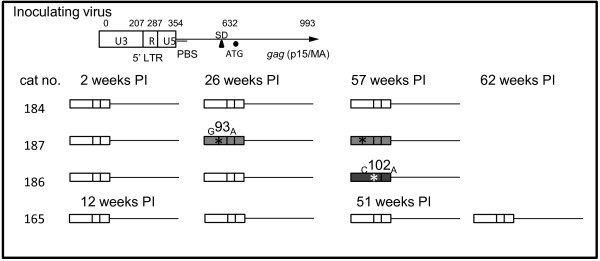
**Stability of the LTR/*gag *sequence in the integrated FIV provirus during the course of infection**. The subgenomic region of the inoculating FIV provirus, shown at the top of the figure, was PCR-amplified from PBMCs of infected cats and analyzed by DNA sequencing. Relative nucleotide positions within the amplified sequence are indicated with PBS denoting the primer binding sequence, SD denoting the splice donor, and ATG marking the start of *gag *translation. Viral sequences derived from PBMCs isolated at different time points from the four different FIV-infected cats are depicted schematically.

**Figure 8:**
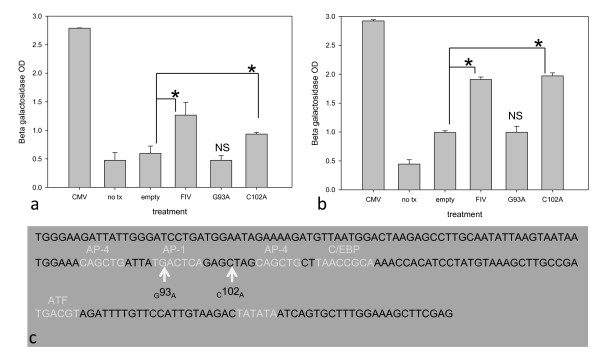
**Point mutations in the FIV-C promoter showing either no effect or abrogating promoter function in an *in vitro *reporter assay**. In an *in vitro *FIV promoter-β galactosidase reporter system, the promoter of the inoculating virus and the _C_102_A _mutated promoter demonstrated strong basal promoter function in (a) feline CRFK and (b) human 293T cells relative to negative controls (no transfection and a promoterless β galactosidase plasmid- no tx and empty, respectively). Cells transfected with a CMV-β galactosidase (CMV) plasmid served as a positive control. Statistical significance is denoted by an asterisk for selected pair wise comparisons (*, p < .05) whereas error bars denote standard deviation. However, cells transfected with the _G_93_A _promoter mutant failed to demonstrate a basal promoter function above the negative controls (not significant, NS). The sequence of the promoter in the U3 region of the inoculating FIV-C virus (c) demonstrates multiple *cis*-acting transcriptional elements (light grey text). The _G_93_A _mutation lies within the putative AP-1 binding site whereas _C_102_A _is located between binding sites (white arrows).

FIV sequence variation was examined in chronically infected cats through the analysis of viral LTR, the *gag *leader sequence and the 5' terminus (361 nt) of *gag *(*matrix*), as shown in Figure 7. This sequenced region captures the encapsidation determinants in addition to intervening sequences of unknown relevance. In HIV, insertion and deletion mutations have been identified within the *gag *leader sequence at or near the primer binding site in both *in vivo *derived samples and *in vitro *passaged virus [36,37]. A G to A mutation (single nucleotide polymorphism; SNP) was identified at position 93 of the viral genome in samples isolated from cat 187 at both 26 weeks and 57 weeks, relative to inoculating virus sequence. A distinct C to A SNP was detected in samples isolated from cat 186 at 57 weeks at position 102. No LTR sequence changes were identified in samples isolated from cats 165 or 184 at any time point (4 samples analyzed for cat 165 and three samples for cat 184). In order to address promoter functionality, the entire LTR of the inoculating virus was cloned and utilized in the construction of an FIV LTR-β galactosidase reporter plasmid (pBlue TOPO, Invitrogen). In addition, this reporter plasmid was mutated to reflect point mutations observed at positions 93 and 102 in U3 of FIV LTR sequences from cats 186 and 187. The FIV promoter demonstrated strong basal activity relative to negative controls based on β galactosidase expression when transfected into human fetal kidney (293T) and feline kidney cells (CRFK, Figure 8). For selected pair wise comparisons in both the feline and human cell lines, the point mutation at position 102 (C to A) resulted in basal promoter expression levels comparable to the consensus FIV LTR, but the mutation at position 93 (G to A) abrogated basal FIV promoter expression to the level of the negative controls. Interestingly, the mutation at position 93 resides within the AP-1 transcription factor binding site, which may explain the observed decrease in promoter function. For each cell line, these experiments were repeated 3 times with similar results.

No sequence differences were identified between the inoculating virus and viral sequences isolated from infected cats at any time point within the *gag *leader/*gag *open reading frame (2, 12, 26, 51, 57 and 62 weeks post infection). In summary, only two SNP mutations were identified in this set of viral sequences isolated from *in vivo *infected PBMCs. PBMCs harvested from two FIV-infected cats (cat 165 and 184) demonstrated viral sequences identical to the inoculating virus throughout the analyzed region (Figure 7). These data are supportive of minimal virus replication within PBMCs.

## Discussion

A previous study described a state of FIV latency in PBMCs in which plasma vRNA was undetectable by quantitative RT-PCR in asymptomatic cats chronically infected with FIV [38]. In another study, a model of FIV latency was produced in cats by mucosal administration of low-dose cell-associated FIV [39]. FIV establishes latency in cell lines *in vitro *[30,40]. Our study, however, is the first comprehensive report of FIV cellular latency in peripheral blood CD4+CD25+ and CD4+CD25- T cells as demonstrated by real-time PCR/RT-PCR, circle junction PCR analyses and sequencing of the LTR and *gag *gene of the integrated FIV provirus over an extended period of time and chronic infection. Notably, although FIV appears to be transcriptionally silent in both resting and activated CD4+ T cells, HIV-1 is thought to be silent only in resting T cells [41], suggesting that some differences exist between FIV and HIV-1 in viral-host cell interactions.

Although the identification of circle junction viral DNA products has been utilized as an indicator of ongoing viral replication in HIV-1 infected individuals, this method is controversial since the circles are stable dead end products that do not support viral replication [32,35]. In fact, HIV-infected elite suppressors, relative to HIV-infected patients on and off ART, have been shown to harbor low levels of integrated HIV DNA and high levels of 2-LTR circles in PBMCs [42]. In our study, 2-LTR CJ were not detectable from uncultured magnetic column-isolated CD4+ T cells; however, *ex vivo *activation of these sorted cells resulted in detectable 2 LTR CJ, consistent with the concept of a productive viral infection. Sequence analysis of FIV 2 LTR CJ amplicons has not been previously reported, and our analysis demonstrated a variety of inserted nucleotides and deletions at the LTR-LTR circle junction. The most common sequence identified (4 of 8 sequenced clones) contained 6 nucleotides of the primer binding site as well as the dinucleotides flanking the U5 and U3 regions. Similar fragments of the primer binding site sequence have been amplified from 2 LTR CJ products cloned from PBMCs of HIV-1 infected individuals [43]. The most common CJ sequence cloned from HIV-1 infected individuals was 5' GTAC [43,44], which represented only 1 of 8 sequences in our data set. Removal of these 4 nucleotides, GTAC, at the center of the circle junction unambiguously generates the sequences of the LTRs found in integrated proviral DNA [44]. Therefore, integration of retroviruses involves removal of the dinucleotides flanking U5 and U3 from each end of the linear viral DNA [44-46]. Two of our clones demonstrated deletions in the U3 region (10 and 122 nucleotides), which were also identified in samples obtained from HIV-1 infected humans, 8 and 110 nucleotide deletions of HIV U3. Indeed, a large proportion of circular HIV-1 DNA molecules in infected cells demonstrate deletions or other mutations at the circle junction [44]. It is possible that 2-LTR circle junction products are preferentially derived from defective linear intermediates. Alternatively, a large proportion of the linear, integrative form of lentiviral DNA has incorrect termini, rendering it unable to integrate [44].

The viral LTR and *gag *leader/*gag matrix *regions derived from freshly isolated PBMCs demonstrated minimal to no sequence variation over time. Although limiting dilution single genome amplification might reveal other, undetected, changes within the sequenced locus, it is unlikely that frequent or large mutations would remain undetected by the sequencing techniques utilized in this study. FIV encapsidation determinants are bipartite, involving a contiguous region of R, U5 and the proximal aspect of the *gag *leader sequence in addition to the first 230 nucleotides of the *gag *matrix gene [47-50]. Interestingly, and in contrast to what is known for other mammalian retroviruses, the region between the major splice donor and *gag *gene has been demonstrated to be dispensable for FIV encapsidation [49,50]. Therefore, a genetic locus within the 3' aspect of the FIV gag leader region is not constrained by encapsidation sequence requirements and has no gene coding function. FIV genetic variation in this region over time therefore seems plausible. The lack of sequence variation in the *gag *leader/*matrix *gene between the inoculating virus and viral isolates obtained from chronically infected cats suggests that genetic constraints, in addition to the encapsidation determinants and tRNA primer binding site, may be present in this region. We found the FIV promoter derived from the inoculating virus to be functional in both feline and human cell lines. Two U3 promoter mutations were identified in two of the cats, at position 93 and 102. Although the mutation at position 102 had no apparent effect on FIV promoter function, the mutation at position 93 abrogated basal promoter expression. Nucleotide position 93 lies within an AP-1 transcription factor binding site while locus 102 is between known binding sites. Although previous studies have demonstrated that deletion of the FIV AP-1 site had negligible effects on virus expression, deletion of multiple *cis*-acting transcriptional elements (including AP-1) has been demonstrated to attenuate FIV [51]. These findings suggest that at least in one cat (187), lentiviral transcriptional attenuation and latency may be associated with mutations within the U3 promoter.

The intermittently detectable vRNA in the PBMC samples may be due to the presence of vRNA-positive monocytic cells within the PBMC samples. However, FIV is capable of infecting other leukocyte subtypes, such as B lymphocytes and CD8+ T cells [1,3,52-54], which could be productively infected and contributing to these intermittently vRNA positive PBMC samples. Viral antigen has been immunohistochemically localized in feline tissues *in situ *within T lymphocytes, macrophages and dendritic cells [55]. In a transmucosal FIV infection model, evidence suggests that within hours of exposure, mucosal dendritic cells and CD3+ T cells are infected and rapidly traffic to systemic lymphoid tissues [56]. Lentivirus-infected monocytes are rarely detected in the blood of animals or people infected with immunodeficiency-inducing lentiviruses, yet tissue macrophages are thought to be a significant reservoir of virus-infected cells *in vivo *[57]. English *et al. *detected FIV provirus in uncultured monocyte-enriched PBMCs in only 1 in 10 asymptomatic cats infected with FIV-NCSU1, a clade A strain [54]. However, some strains of virus are apparently monocytotropic *in vivo*, and this property may correlate with virulence [57]. Importantly, data from this current study provide additional support for FIV-C-Pgmr as a monocytotropic strain.

Although low copy numbers of vRNA were intermittently identified in freshly isolated PBMCs and monocytes from FIV-infected cats, no vRNA was detected in plasma by sensitive real-time PCR assays at any time point after 44 weeks. This finding could be explained in several ways. It may be due to an insufficient sensitivity of the real-time RT-PCR assay used in this study. The threshold of detection for the vRNA assay was approximately 80 copies vRNA per ml of plasma; therefore, low levels of viremia could exist that remain undetectable through the assays employed in this study. The chronically infected cats may be analogous to HIV-1 infected patients on antiretroviral therapy, thought to have an occult viremia of between 1 and 50 copies of vRNA per ml of plasma that is not detectable by standard real-time RT-PCR assays [27,58]. Alternatively, rare copies of gag vRNA detected within the freshly isolated monocytes and PBMCs may not be translated into viral proteins and/or assembled into releasable virions due to an unidentified post-transcriptional blockade mechanism (*e.g*. inhibition of viral translation, assembly or release).

For HIV-1 infected individuals on ART, cells harboring defective integrated proviruses are found at frequencies 100-600-fold greater than cells with replication competent genomes [59,60]. Defective genomes within PBMCs and CD4+ T cells isolated from chronically infected cats were not investigated in this study. However, productive viral infection through *ex vivo *culture and activation of latently infected cells from chronically infected cats were demonstrated by multiple approaches. These results strongly suggest that at least a portion of the latently infected PBMCs harbor integrated replication competent FIV proviruses that are capable of producing infectious virions through mechanism(s) of transcriptional activation.

## Conclusions

Through the isolation of latently-infected leukocyte subtypes and access to tissues (*via *both surgical biopsy and necropsy), chronic FIV infection of cats may provide a useful animal model for the elucidation of *in vivo *mechanisms of lentiviral latency. As such, the FIV-infected cat in the asymptomatic phase is proposed as a model of peripheral CD4+ T cell latency. Importantly, mechanisms of transcriptional repression identified in this animal model of lentiviral latency may reveal analogous mechanisms of persistence in ART-treated HIV-infected human patients.

## Methods

### Animals and virus

Six FIV SPF kittens were obtained from the breeding colony of the Feline Nutrition Laboratory, University California at Davis (UC Davis). The kittens ranged in age from 4 to 5 months and were housed in the Feline Research Laboratory (FRL) of the Center for Companion Animal Health (CCAH), UC Davis. Husbandry care was provided by staff of the CCAH under the supervision of the Center for Laboratory Animal Services, UC Davis. The study protocol was approved by the UC Davis Institutional Animal Care and Use Committee.

A single 5 1/2 month old male kitten (165) was inoculated with one ml FIV-C-Pgmr viral inoculum (~10^5 ^TCID_50_) in the musculature of the left caudal thigh. The FIV-C-Pgmr isolate was provided by Drs. E. Hoover and N. Pedersen. This kitten was closely monitored for clinical signs of FIV-associated immune deficiency weekly and peripheral blood was assayed for complete blood count, FIV antibody by ELISA (SNAP FIV/FeLV Combo test, IDEXX Laboratories, Westbrook, Maine), FIV p24 antigen by ELISA, and changes in CD4+ and CD8+ frequency by flow cytometry every two weeks. Viral loads in blood were tested by real-time RT-PCR assays measuring copy numbers of cellular FIV vDNA and plasma FIV RNA. Six weeks post-inoculation, plasma was harvested from the FIV-infected kitten, clarified, and used to infect SPF feline PBMCs *in vitro*. Eleven days post-infection, clarified supernatant from plasma-inoculated PBMC cultures was determined to contain 7.5 × 10^8 ^viral RNA copies/ml by real-time PCR. Three 4-5 month old kittens (cats 184, 186 and 187, all males) were next inoculated with culture supernatant (1 ml; 7.5 × 10^7 ^viral RNA copies, 1:10 dilution of virus stock in sterile media) in the left caudal thigh musculature, as described above. Two control kittens (183 and 185) were mock-inoculated with 1 ml of sterile culture media. Kittens were housed, monitored for clinical illness by physical examinations, and sampled for blood by venipuncture every two to four weeks up to 112 weeks after inoculation.

### CD4+ lymphocyte and monocyte purification

Feline whole blood was collected by jugular venipuncture and PBMCs were harvested by density gradient centrifugation through Ficoll-Hypaque (Sigma, St. Louis, Mo.). Isolated PBMCs were resuspended and separated through a combination of magnetic-bead columns (Miltenyi Biotec Inc., Auburn, CA) and flow cytometric cell sorting. Briefly, cells were enumerated using an automated cell counter (Coulter A^C^T diff, Beckman Coulter) and approximately 2 x10^7 ^were used for primary antibody (Ab) binding. PBMCs were initially depleted of monocytes, B cells and granulocytes via an LD column (MACS Separation Columns, Miltenyi Biotec Inc., Auburn, CA) and a cocktail of mouse anti-feline antibodies: anti-CD11b (clone CA16.3E10-IgG1), anti-CD8α (clone FE1.10E9-IgG1), anti-CD21 (clone CA2.1D6-IgG1), (antibodies provided by Peter Moore, UC Davis) and goat-anti mouse IgG-microbeads (Miltenyi Biotec) according to the manufacturers' instructions. The flow-through cell fraction was treated with mouse anti-feline CD25 monoclonal antibody (clone 9F23; gift of Koichi Ohno, University of Tokyo) and applied onto a MS column (MACS Separation Columns, Miltenyi Biotec Inc) along with goat-anti mouse IgG-microbeads (Miltenyi Biotec). Column-sorted cells were treated with a blocking antibody (1 mg/ml, CA2.1D6, FHCRC Biologics). Anti CD4-FITC antibodies (FE1.7B12, P. Moore, UC Davis) were used to label both the cells retained by the MS column and the flow-through fractions. Monocytes were isolated from the cell fraction retained from the LD column. A conjugated secondary antibody was added to these retained cells (mouse anti-human CD-14-ALEXA Fluor 647; AbD Serotec). PBMCs labeled with ALEXA and FITC-conjugated secondary antibodies were secondarily sorted and enumerated by flow cytometry (MoFlo Cell Sorter, Cytomation Inc., Fort Collins, CO) at the UC Davis Flow Cytometry Shared Resource. Cells were gated on size (forward scatter/sidescatter), live cells (propidium iodide exclusion), single cells (pulse width) and fluorochrome expression (ALEXA or FITC). Typical yields for monocytes, CD4+CD25+ and CD4+CD25-, were 2.5 × 10^5 ^cells, 2.4 × 10^5 ^and 1.7 × 10^6^, respectively. The purity of all three sorted cell populations was determined to be approximately 99%, as determined by CD14 or CD4 expression (monocytes and CD4+ T lymphocytes, respectively).

For virus rescue assays from latently infected CD4+ T cells, a modified protocol was followed for isolating viable CD4+ T cells. In this modified protocol, PBMCs from both FIV-infected and uninfected animals were depleted using an LD column (Miltenyi Biotec) as described above. The flow-through cell fraction was treated with anti CD4 antibodies (FE1.7B12, P. Moore, UC Davis) along with goat-anti mouse IgG-microbeads (Miltenyi Biotec). Labeled cells were then applied to a second LD column and retained cells were immediately cultured *ex vivo*. Typical yields of CD4+ T cells were 6 × 10^6 ^cells while cell purity was determined to be 97% CD4+ T cells, with approximately 0.1% contaminating CD14+ cells and less than 3% CD4- CD14- cells.

### Hematologic assessment of cats

Complete blood cell counts were performed on Coulter counter (Coulter ACT diff2, Beckman Coulter, Fullerton, CA) and differentials were performed on cytologic smears of whole blood. PBMCs isolated by Ficoll Hypaque gradient centrifugation were phenotyped for CD4+ and CD8+ lymphocyte frequency by flow cytometry using anti-feline CD4 (FE1.7B12) and anti-feline CD8α (FE1.10E9). Secondary antibody, fluorescein-conjugated horse anti-mouse IgG (Vector Laboratories, Inc., Burlingame, CA) was utilized for detection. Samples were analyzed on a FACScan instrument (Becton Dickinson, San Jose, CA) using FloJo v8.6.3 flow cytometry analysis software (Tree Star, Ashland, OR). Absolute CD4+ and CD8+ cell counts in blood were derived from absolute lymphocyte counts and subset frequencies determined by flow cytometry.

### Real-time PCR assays

Cell-associated RNA and DNA were co-isolated utilizing a commercial kit (AllPrep DNA/RNA Mini Kit, Qiagen, Valencia, CA) while plasma or culture media-associated vRNA was isolated using a different commercial kit (QIAamp Viral RNA Mini Kit, Qiagen) according to manufacturers' instructions. DNase treatment of isolated RNA was accomplished with TURBO DNase (Ambion, Austin, TX). RNA was reverse transcribed into cDNA with the OriGene 1^st ^Strand cDNA Synthesis System for Quantitative RT-PCR (Origene, Rockville, MD). A control reaction excluding reverse transcriptase was included for each sample. Real-time PCR assays were performed in triplicate with Real Mastermix SYBR Rox (5 Prime, Gaithersburg, MD) on an Applied Biosystems 7300 Real-time PCR System and subsequently analyzed with the 7300 system software (Applied Biosystems, Carlsbad, CA). Real-time PCR and RT-PCR assays to quantify viral nucleic acids used primers based on FIV-C-Pgmr *gag *sequence (Table 1). The PCR annealing temperature was optimized using a gradient PCR system (Eppendorf Mastercycler). Quantification of plasma FIV RNA copy number was based on a standard curve generated from viral transcripts prepared by *in vitro *transcription of a plasmid (pCR2.1, Invitrogen) containing a 101 nucleotide-long FIV-C-Pgmr *gag *amplicon.

For assay of cell- associated vDNA and vRNA, real-time PCR assays of feline GAPDH were included to normalize input nucleic acid concentration. Because there are two allelic copies of the housekeeping gene GAPDH per cellular genome [61], viral gag DNA copy number was normalized to 1 × 10^6 ^copies of GAPDH (determined from the same sample) which was expressed as DNA copy number per 5 × 10^5 ^cells (copies GAPDH/2 = number of cells). For RT-PCR, a similarly derived standard curve specific for feline GAPDH transcripts was utilized. Viral RNA copy number was normalized to 1 × 10^6 ^copies cellular GAPDH RNA, performed on the same RNA sample in parallel. The cycling conditions for GAPDH assay were as follows: 50°C for 2 minutes, 95°C for 2 minutes followed by 40 cycles of 95°C for 15 sec and 60°C for 30 sec and a final elongation step at 72°C for 5 minutes. The cycling conditions for the FIV gag assay were as follows: 50°C for 2 minutes, 95°C for 2 minutes followed by 40 cycles of 95°C for 15 sec, 58°C for 30 sec, 68°C for 30 sec and a final elongation step at 72°C for 5 minutes. The cycling conditions for 2 LTR circle junction assay were as follows: 50°C for 2 minutes, 95°C for 2 minutes followed by 40 cycles of 95°C for 15 sec, 56°C for 30 sec and 72°C for 30 seconds. All real-time PCR assays were followed with a dissociation step (melt curve).

### Standard PCR assays

Standard PCR was performed with Platinum Taq DNA polymerase (Invitrogen) on an Eppendorf Mastercycler (Eppendorf). The cycling conditions for PCR amplification of LTR sequences, *gag *leader/*gag *orf sequences, and feline 18s ribosomal RNA were as follows: 95°C for 2 minutes followed by 40 cycles of 95°C for 15 sec, 56°C for 30 sec, 72°C for 30 sec and a final elongation step at 72°C for 5 minutes. Circle junction PCR (primer sets A and B) was performed using the following reaction conditions: 95°C for 5 minutes followed by 30 cycles of 95°C for 30 sec, 58°C for 60 sec, 72°C for 120 sec and a final elongation step at 72°C for 5 minutes. PCR products were analyzed on 1-2% agarose gels, stained with ethidium bromide for visualization.

### Primers for PCR

Primers were designed based on GenBank sequences and sequences derived from the FIV-C-Pgmr viral inoculum. Real-time PCR primers (FIV gag and feline GAPDH) were optimized using primer design software (Integrated DNA technologies Inc. OligoAnalyzer- http://www.idtdna.com/analyzer/Applications/OligoAnalyzer/) and were synthesized by a commercial vendor (IDT, Coralville, IA). Two LTR CJ primers were based on cloned sequences. Primer pairs are further described by name, sequence, FIV genomic location and assay type in Table 1. Real-time PCR primers were utilized as follows: feline GAPDH (Feline _GAPDH for _and Feline _GAPDH rev_), 2-LTR circle junction/CJ PCR C (FIV _U5 for _and FIV _U3 rev_), and FIV gag (FIV _QT gag for _and FIV _QT gag rev_). Standard PCR primers were utilized as follows: circle junction PCR A (FIV _env for _and FIV _gag rev_), circle junction PCR B (FIV _env for _and FIV _gag circle rev3_), LTR (FIV _env for _and FIV _U5 rev_), *gag *leader/*gag *orf (FIV_Gag leader for _and Gag_circle rev_) and Feline 18s rRNA (18s _rRNA for _and 18s _rRNA rev_).

### Nucleotide sequencing and analysis

A proviral subgenomic fragment containing the long terminal repeat (LTR) through the first 637 nucleotides of the FIV leader and *gag *gene (nt 1-993) were PCR amplified from genomic DNA isolated from PBMCs of each FIV-infected cat and then sequenced. Viral sequences were determined for different time points including acute infection (2 weeks PI), early chronic infection (21-26 weeks PI) and chronic infection (51-62 weeks PI). Nucleotide sequence of the FIV-C-Pgmr virus inoculum was determined from cDNA generated from inoculum viral RNA. Nucleotide sequence of inoculum cDNA was based on molecularly cloned PCR products (pCR2.1, TA cloning system, Invitrogen, Carlsbad, CA). Nucleotide sequences were aligned and compared using the *AlignX *function of Vector NTI software (Invitrogen, Carlsbad, CA).

### Virus rescue assays

PBMCs were isolated from blood samples by Ficoll-Hypaque (Sigma-Aldrich) density centrifugation for both FIV-infected and uninfected cats at 50 weeks post infection and cultivated using previously described protocols [34] with the modification that cells were also cultured with 0.1 μg PMA/ml (Sigma-Aldrich) in addition to 5 μg Con A (ThermoFisher Scientific) per ml for activation of viral gene expression. PBMC cultures were provided with fresh media on day 8. PBMCs harvested on day 10 were processed for genomic DNA to test for viral DNA loads by real-time PCR assays based on *gag *sequence. Similarly, PBMC culture supernatants were collected on day 10 to assay for viral RNA by real-time RT-PCR and to assess for infectivity on virus-naïve SPF feline PBMCs cultured by standard protocols [34]. Magnetic column-Isolated viable CD4+T cells were cultured *ex vivo *as described for PBMCs at a concentration of 2 million cells/ml media. The media were exchanged for fresh media every 5 days. On days 3 or 14 of culture, CD4+ T cells were co-cultured with FIV-negative feline PBMCs or CD4+ T cells for 5, 6 or 10 additional days. *Ex vivo*-cultured cells were processed for nucleic acids and assayed by PCR as described above for PBMCs.

For virus infectivity assays, approximately 9 × 10^6 ^SPF PBMC cultures were inoculated with 0.5 mL of clarified supernatant harvested from PBMC cultures derived from FIV-infected or uninfected (control) cats. On day 7 of culture, cells were harvested and processed for RNA and DNA using a commercial kit (Qiagen DNA/RNA minikit, Qiagen). PBMC culture supernatants were also harvested and tested for vRNA using real-time RT-PCR as described in the Methods section for PCR assays.

### FIV promoter-reporter assay

DNA was isolated from PBMCs infected with the inoculating FIV-C-Pgmr (QIAamp DNA Minikit, Qiagen). The proviral LTR was amplified via PCR (FIV _env for _and FIV _U5 rev_) and cloned into a β galactosidase expression plasmid (pLTR blue, derived from pBlue TOPO, Invitrogen). The sequence of the proviral LTR was confirmed by sequencing the insert. To replicate the two point mutations identified in the FIV U3 promoter of cats 186 and 187, targeted point mutation of pLTR blue was performed with a commercial mutatagensis kit (Quikchange Lightening Site-Directed Mutagenesis Kit, Agilent Technologies, La Jolla, CA) according to the kit instructions, and using primers designed on the manufacturer's website http://www.stratagene.com/qcprimerdesign. The resulting plasmids were sequenced and found to be identical to pLTR blue, with the exception of a G to A substitution at position 93 within U3 (pLTR_G_93_A _blue), or a C to A substitution at position 102 within U3 (pLTR_C_102_A _blue). CRFK cells (ATCC, Manassas, VA), and 293T cells (courtesy of Washington State University) were transfected with pLTR blue, pLTR_G_93_A _blue, pLTR_C_102_A _blue, control plasmids, or no plasmid (Lipofectamine 2000 Reagent, Invitrogen) according to the Lipofectamine protocol. Control plasmids consisted of a positive control plasmid with the CMV promoter upstream of the β galactosidase gene (pcDNA 3.1D/V5-His/lacZ, Invitrogen) and a promoterless β galactosidase plasmid (negative control). Transfected cells were harvested 48 hours later and a β galactosidase assay was performed in triplicate with cell lysates according to standard protocols [62]. Relative β galactosidase activity was determined by a spectrophotometer; assays were repeated three times.

### Statistical analysis

Data are presented as the mean of three or more values with the standard deviation displayed as error bars. An analysis of variance was performed (ANOVA) on each data set. Where global differences were identified, the Tukey-Kramer Multiple Comparisons Test was utilized for pair-wise comparisons of the mean responses between treatment groups. Error bars denote standard deviation. A P value < 0.05 was considered to be statistically significant. Statistics were performed with InStat software (GraphPad Software Inc., La Jolla, CA).

## List of abbreviations

LTR: long terminal repeat; FIV: feline immunodeficiency virus; PBMC: peripheral blood mononuclear cell; HIV-1: human immunodeficiency virus-1; ART: anti-retroviral therapy; SPF: specific pathogen-free; RT: reverse transcription; PCR: polymerase chain reaction; CJ: circle junction; Con A: concanavalin A; PMA: phorbol myristate acetate.

## Competing interests

The authors declare that they have no competing interests.

## Authors' contributions

BM designed the overall study and experiments, interpreted all of the data and wrote all the manuscript drafts. NV performed much of the early experiments (year 1), optimized the methods and assisted in data analysis. CH and DC performed much of the later experiments (year 2), assisted in data analysis and drafting of the manuscript. DC performed the β-galactosidase assays. SM optimized and performed the CD4+ T cell isolation along with the 2-LTR CJ analyses, LTR mutagenesis strategy and data interpretation. PM assisted in the design and optimization of the leukocyte isolation strategy. PAL and EES assisted in the overall study design and analysis of experiments and edited all the manuscript drafts. All authors read and approved the final manuscript.
